# The Implications of the Diving Response in Reducing Panic Symptoms

**DOI:** 10.3389/fpsyt.2021.784884

**Published:** 2021-11-29

**Authors:** Peter Kyriakoulis, Michael Kyrios, Antonio Egidio Nardi, Rafael C. Freire, Mark Schier

**Affiliations:** ^1^School of Health Sciences, Swinburne University of Technology, Hawthorn, VIC, Australia; ^2^College of Education, Psychology & Social Work, Orama Institute for Mental Health & Wellbeing, Flinders University, Adelaide, SA, Australia; ^3^Institute of Psychiatry-Federal University of Rio De Janeiro, Rio De Janeiro, Brazil; ^4^Department of Psychiatry and Centre for Neuroscience Studies, Queen's University, Kingston, ON, Canada; ^5^School of Health, Swinburne University, Hawthorn, VIC, Australia

**Keywords:** panic disorder, diving response, cold facial immersion, CO_2_ sensitivity, anxiety

## Abstract

Increased CO_2_ sensitivity is common in panic disorder (PD) patients. Free divers who are known for their exceptional breathing control have lower CO_2_ sensitivity due to training effects. This study aimed to investigate the immediate effects of cold facial immersion (CFI), breath holding and CO_2_ challenges on panic symptoms. Healthy participants and patients with PD were subjected to four experimental conditions in a randomly assigned order. The four conditions were (a) breath-holding (BH), (b) CFI for 30 s, (c) CO_2_ challenge, and (d) CO_2_ challenge followed by CFI. Participants completed a battery of psychological measures, and physiological data (heart rate and respiration rate) were collected following each experimental condition. Participants with PD were unable to hold their breath for as long as normal controls; however, this finding was not significant, potentially due to a small sample size. Significant reductions in both physiological and cognitive symptoms of panic were noted in the clinical group following the CFI task. As hypothesized, the CFI task exerted demonstrable anxiolytic effects in the clinical group in this study by reducing heart rate significantly and lessening self-reported symptoms of anxiety and panic. This outcome demonstrates the promise of the CFI task for clinical applications.

## Introduction

According to DSM-5, panic disorder (PD) is a severe and persistent anxiety disorder characterized by spontaneous and recurrent panic attacks (PAs) (1, 2). PD sufferers exhibit irregularities in respiratory rhythms, predominantly a thoracic pattern of breathing, abnormal variability, and irregularity in breathing (3, 4). In addition, several respiratory symptoms have been associated with PD, including air hunger, dyspnea, rapid breathing, and elevated heart rate (5).

CO_2_ hypersensitivity theory proposes that PD sufferers have a lower physiological threshold for detecting CO_2_ levels (5). It is proposed the existence of an evolved suffocation alarm system that helps the brain monitor useful air, consistent with the lowered threshold for detecting CO_2_ levels (6). According to this model, PAs occur when the brain mistakenly detects a lack of useful air (increased CO_2_), triggering the suffocation alarm system. This maladaptive response makes PD sufferers vulnerable to “false suffocation alarms,” specifically PAs.

For PD sufferers, the CO_2_ challenge has a more exaggerated response than normal controls, inducing a sharp and transitory rise in anxiety that has been compared to a PA (6–12). However, studies comparing PD patients with other anxiety disorders and normal controls have yielded mixed results. For instance, breath-hold times were lower in PD sufferers than in normal controls but no lower than in sufferers of other anxiety disorders (13). Meanwhile, PD patients exhibited shorter breath-hold times than patients with other anxiety disorders (14). Furthermore, PD sufferers experienced elevated physiological reactivity to the breath-hold challenge (15). Their findings support the false suffocation alarm system (6) present in PD sufferers.

More recently, research combining neuroimaging and panic provocation challenges provided further scientific insights into interoceptive sensory triggers and potential neural mechanisms that underlie spontaneous PAs (16). The role of acid-base and chemosensory mechanisms has been identified as an important internal homeostatic trigger for PAs. A large body of research proposes that the fear network, an association of fear circuits in the brain comprising the amygdala, hippocampus, medial prefrontal cortex, brain stem projections and insula, may be abnormally sensitive in PD patients and particularly sensitive to homeostatic changes (17, 18). More specifically, within the cycle of panic, a sensitivity in detecting threats to homeostasis, acidosis, may sensitize fear-arousal-stress regulatory circuits to other triggers leading to PD.

A research proposed the notion of a continuous trait based on one's physiological response to increasing CO_2_ levels, where PD patients characterized by hypersensitivity to CO_2_ are positioned at one end of the spectrum (19). At the other end are those individuals with low sensitivity to CO_2_ increases. These include free divers (20, 21). Free divers are known for their exceptional breathing control and lower ventilatory response to CO_2_, which has been related to training and diving experience (22).

Free divers practice the sport of diving on one breath and draw on a range of breathing techniques to assist them in attaining greater depths underwater, with some able to hold their breath underwater for 10 min (23). The extraordinary breath-hold ability found in free divers can be explained by an evolved physiological response that helps mammals stay underwater for long periods of time, known as the diving response (DR).

The DR is a physiological reflex that optimizes respiration, allowing humans to endure a lack of oxygen underwater. It is activated by apnea, also known as breath holding, and CFI (stimulation of the cold facial receptors with water) (24, 25). Research indicates that facial cold receptors are more strongly stimulated by immersion in water at temperatures ranging from 10 to 15°C (26).

The physiological adaptations associated with the DR include a decrease in heart rate (bradycardia) and cardiac output, vascular constriction, reduced blood flow to peripheral capillary beds and increased blood pressure. Cardiovascular adjustments and their pronounced bradycardic effect serve as an oxygen-conserving reflex that aims to maintain life during asphyxia by enhancing blood flow to vital organs (heart, brain, and lungs) (27–30). In many respects, the physiological adjustments comprising the nervous, cardiovascular and respiratory systems that act to promote oxygen conservation during the DR are the opposite of those triggered in PAs.

Long-term training of free diving is associated with several physiological adaptations, including a more pronounced DR, greater lung volume, lung oxygen, and carbon dioxide stores (25, 31). Trained breath-hold divers will endure the human DR during a breath hold until PaO_2_ has fallen to 35 mmHg and PaCO_2_ has increased to 50 mmHg, whereas non-divers when engaging in breath-hold activity can generally reduce their PaO_2_ as low as 60 mmHg and their PaCO_2_ as high as 45 mmHg (32). Further support that breath-hold training builds greater tolerance to CO_2_ is found in trained synchronized swimmers who can sustain a normoxic breath hold for approximately twice the breath-hold time compared to non-diving controls (33). It was demonstrated that 2 weeks of daily apneic (breath-hold) training increased both the DR and the duration of breath-hold (34).

Cold water facial immersion is superior in reducing heart rate when compared to immersions of other body parts and that the water temperature is a significant stimulus for driving the DR (35): the greater the difference between ambient air temperature and water temperature, the more dramatic the bradycardic response will be (36). Given that there is a higher density of receptors in the ophthalmic region of the trigeminal nerve that includes the eyes, forehead, and nose, there is greater sensitivity to cold water when the face is fully submerged in water (37).

One of the cardinal features of PD when fear is elicited is a dysregulated ANS, characterized by sympathetic nervous system (SNS) arousal. It has been established that effecting reductions in heart rate can provide substantial acute symptomatic relief for persons in panic states. Frequently treatments are ineffective and costly, hence greater knowledge of the underlying pathophysiology of PD is required to assist with the development of effective treatments (38). PD patients may be able to benefit from simple and practical treatments aimed at regulating the ANS and reversing the fear response. Hence, this study explores whether the DR, activated through breath holding and CFI and its consequential bradycardic effect, is able to reduce the psychophysiological fear response associated with panic.

The current study investigated the immediate effects of breath holding and carbon dioxide challenge. The specific objectives are to (1) examine preliminary data on the short-term effects of CFI on physiological and psychological panic symptoms induced by respiratory challenges and (2) compare a group of PD participants with a control group to examine the magnitude of differences between these groups. This study is the first attempt to examine the relationship of CO_2_ sensitivity threshold and panic symptoms in order to better understand possible applications of the DR and CFI to the treatment of panic symptoms.

## Methods

### Participants and Sampling

Investigations were carried out with 32 participants: 16 patients with a primary diagnosis of PD with or without agoraphobia (DSM-5) (clinical group, or group 1) and 16 normal controls who did not meet the criteria for PD or mental illness (control group, or group 2). Of these, 6 were male, and 26 were female. The clinical group comprised 1 male and 15 females, and the control group comprised 5 males and 11 females. The participants in the clinical group had an average age of 36.43 years (*SD* = 2.82), and the participants in the control group had an average age of 29.06 years (*SD* = 1.79). The cohort differences are reported in the results section.

Health screening assessments were carried out by a medical doctor (at Swinburne University) to establish medical eligibility to undergo the CO_2_ challenge. The Mini-International Neuropsychiatric Interview (MINI) is a short structured diagnostic interview used to make diagnoses of Axis I disorders (DSM-IV) has demonstrated high reliability and validity (39). It was used to screen psychological disorders within the exclusion criteria and identify individuals with PD (DSM-IV). Exclusion criteria for both groups included psychotic disorders, substance abuse, prescription medication, habitual use of benzodiazepines, known allergies to latex, asthma or respiratory problems, cardiovascular problems, hypertension, hypotension, pregnancy, and cerebrovascular problems including epilepsy and organic brain disorder. Finally, other comorbidities to Axis I mental health disorders (DSM-IV) were excluded, along with those with first degree biological relatives with PD. The Panic Group was also assessed with the structured clinical interview (SCID-I) for the DSM-IV Axis I Disorders module for Panic Disorder and Agoraphobia (SCID-I) (40). The SCID-I is a comprehensive structured interview for diagnosis of psychiatric disorders according to DSM-IV criteria (41).

### Physiological Measures

A compact physiological monitoring system (Zephyr Bioharness) featuring a chest strap and external multirecording and monitoring device was used to measure heart rate, posture and respiration rate. Participants were connected to Zephyr Bioharness and measured throughout the experimental study. Participants' breath-hold ability was also measured during the experimental phase and included breath-hold ability after exhalation and breath-hold ability following maximum inhalation. All participants undertook all four conditions of the experimental study while wearing the chest strap connected to the Zephyr Bioharness, which was connected via Bluetooth to a multirecording and monitoring device (PowerLab). PowerLab version 7.0 data acquisition and analysis software were used as a multirecording device, and recorded data were sampled at a frequency of 60 Hz (60 samples per second).

### Research Design

**Figure 1:**
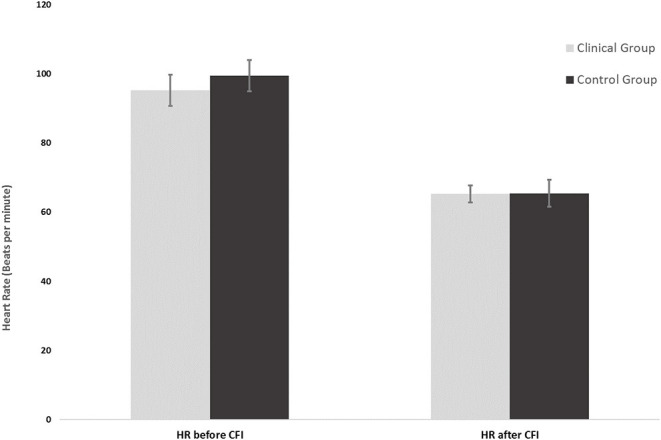
Mean heart rates of the clinical group (*n* = 16) and control group (*n* = 16) before and after CFI.

Participants took part in four experimental conditions, where the order of conditions was randomly assigned. The experiment was conducted in a quiet clinical office. Figure 1 displays the experimental conditions and data collection.

#### Condition A: Breath-Hold Challenge

Participants wore a nose clip and were instructed to hold their breath for as long as possible, following an exhalation. Upon completion of this breath-hold challenge, they were given a 1-min rest period before being instructed again to hold their breath after taking a maximum inhalation. Physiological measures including breath-hold times, heart rate (HR) and respiration rate (RR) were recorded.

#### Condition B: CFI With 30 s of Apnea (CFI Challenge)

A sterilized container was filled with water and placed on the left side of a medical bed, which was cleared of any other apparatus. Sufficient space around the water container was allowed for participants to rest their arms on both sides of the container while completing the task. Participants were instructed to take a maximal inhalation and then immerse their entire face in the water (including the forehead) while holding their breath for 30 s. Participants were informed that they could terminate the exercise at any time if they felt discomfort or a strong urge to breathe. The researcher counted out aloud in intervals of 5 s until 30 s was reached, at which point participants were asked to lift their face from the water. The ambient air temperature was controlled at 22°C, and the water temperature was maintained between 7 and 12°C.

#### Condition C: CO_2_ Challenge

A single-breath CO_2_challenge was used to evoke and rate participants' anxious response to the challenge. Participants were informed before the challenge that they would inhale a single breath of a CO_2_ mix (comprising concentrations of 35% CO_2_ and 65% O_2_) via a Douglas bag and that while the procedure is completely safe, it may evoke some transient breathlessness or discomfort. Participants were instructed to wear a nose clip and exhale the air from the lungs before they inhaled a single maximum inhalation of the premixed air in the Douglas bag. The 35% CO_2_ challenge is considered a valid procedure if the participant inhales at least 80% of their vital capacity (66). Participants were asked to hold their breath before exhaling for 4 s while the researcher counted aloud from 1 to 4. Participants were then asked to mark on the Visual Analog Scale (VAS) how anxious they felt. The VAS was a scale anchored by 0 (no anxiety at all) to 10 (worst anxiety). The researcher also rated (on a similar VAS) how anxious the participant looked following the CO_2_ challenge for concurrent external validity. Participants then sat down and completed the battery of psychological assessments.

#### Condition D: CO_2_ + CFI Challenge

CO_2_ participants were then asked to perform the CFI task (as per Condition B) as soon as they felt comfortable enough. Once complete, both the participant and researcher independently rated the level of anxiety on the VAS. The participant was then asked to complete the battery of psychological measures.

### Data Cleaning

Prior to statistical analysis, all variables were assessed for the presence of missing data and univariate outliers. No outliers were identified in the clinical group or control group of participants, and no missing data were identified in any of the psychological measures or physiological measures collected during the premeasure phase or experimental phase. Non-parametric analyses were conducted in cases where data did not adequately meet the assumptions of normality and could not be transformed to normalize their distributions according to recommended procedures (42).

### Statistical Analyses

Physiological data, including RR and HR data, satisfied the assumptions for parametric analysis. Hence, *t*-tests and ANOVA analyses were used to investigate the differences between the clinical group and control on the experimental conditions. Examination of the scores across all self-report psychological measures taken at pretest, after Condition C (CO_2_), and after Condition D (CFI plus CO_2_), including Acute Panic Inventory (API), Anxiety Sensitivity Inventory (ASI), Beck Anxiety Inventory (BAI), Panic Attack Cognitions Questionnaire (PACQ), State Trait Anxiety Inventory (STAI), Visual Analog Scale—Researcher (VAS-R), and Visual Analog Scale—Participant (VAS-P), were not normally distributed; therefore, non-parametric tests were utilized. Spearman's correlation coefficient was used to investigate the relationships between psychological measures. Friedman's test was used to examine differences in the psychological measures collected across the experimental conditions. The Statistical Package for Social Sciences version 22.0 was used for all analyses. The alpha level was set at 0.05 for all parametric analyses. A Bonferroni adjustment was performed for parametric analyses, resulting in a significance threshold of *p* < 0.016.

## Results

The analysis of this study is presented in two sections. The first section presents the comparison of demographic details between groups and correlations of the pretest measures. The second section presents analyses of the results.

### Participant Demographics and Correlations Between Pretest Measures

Age was significantly higher (*p* = < 0.05) in the clinical group (*M* = 36.4 years; *SD* = 11.3) than in the control group (*M* = 29.1 years; *SD* = 7.2). Tables 1, 2 provide the means on categorical and continuous demographic variables for the clinical and control groups. No significant differences were found between the groups. Although the groups were matched statistically, there were apparent differences in gender and education that may not have been significant due to the small sample size.

**Table 1:** Demographic information: categorical variables.

		**Clinical**		**Control**	
**Variable**	***Fisher's z***	***N***	***%***	***N***	***%***
**Gender**	*p =* 0.172				
Male		1	6.30	5	31.30
Female		15	93.70	11	68.70
**Education level**	*p =* 0.156				
No university degree		11	69.00	6	37.60
University degree		5	31.00	10	62.40
**Employment status**	*p =* 0.479				
Employed		10	62.50	7	43.80
Unemployed/student		6	37.50	9	56.30
**Smoking**	*p =* 1.000				
Yes		2	12.50	3	18.80
No		14	87.50	13	81.20
**Drinking**	*p =* 1.000				
Yes		13	81.30	14	87.50
No		3	18.80	2	12.50
**Physical fitness**	*p =* 1.000				
Poor/fair		7	43.75	7	43.75
Good/very good		9	56.25	9	56.25
**Physical activity at work**	*p =* 0.252				
Sedentary		9	56.30	13	68.80
Non-sedentary		7	43.70	3	31.20
**Weekly physical exercise**	*p* = 1.000				
<3 h		12	75.00	12	75.00
>3 h		4	25.00	4	25.00
**Weekly cycling**	*p* = 1.000				
None		12	75.00	13	81.25
Some		4	25.00	3	18.75
**Weekly walking**	*p =* 0.242				
<3 h		8	50.00	12	75.00
>3 h		8	50.00	4	25.00
**Weekly home duties**	*p* = 0.273				
<3 h		4	25.00	8	50.00
>3 h		12	75.00	8	50.00
**Weekly gardening**	*p* = 1.000				
None		11	68.75	10	62.50
Some		5	31.25	6	37.50
**Walking pace**	*p* = 1.000				
Slow/steady, average		4	25.00	5	31.25
Brisk pace/fast (>6 km/h)		12	75.00	11	68.75

*N = 32 (group 1: n = 16; group 2: n = 16). Fisher's exact test (2-sided)*.

**Table 2:** Demographic information: continuous variables.

	**Clinical**		**Control**	
	***M***	***SD***	***M***	***SD***
Water comfort (Mann-Whitney, *p =* 0.809) (0 = Not at all comfortable−10 = Very comfortable)	7.31	2.68	7.94	1.53
Average no. of glasses per week (Mann-whitney, *p =* 0.423)	3.00	2.88	2.00	1.63
# of Push-ups (Mann-Whitney, *p =* 0.140)	9.40	8.32	16.81	14.19
Height (Mann-whitney, *p =* 0.468)	167.25	7.46	169.12	8.30
Weight (Mann-whitney, *p =* 0.564)	74.22	17.97	70.47	17.02

*p-values (2-sided) are based on the Mann-Whitney test*.

The Spearman rank-order correlation coefficient was used as a non-parametric measure to determine the strength and direction of association that exists between the premeasure assessments. The magnitude of the relationship was determined by the following correlation coefficients: low (0–0.3), moderate (0.4–0.7), and high (≥ 0.8) magnitude of correlations. Table 3 provides correlations between premeasures.

**Table 3:** Correlations between pre-measure assessments.

	**API**	**CESD**	**DIS**	**BAI**	**ASI**	**STAI T**	**STAI S**	**PACQ**
API	–	0.64^**^	0.27	0.69	0.60	0.61	0.60	0.60
CESD		–	0.18	0.71^**^	0.84^**^	0.93^**^	0.83^**^	0.74^**^
DIS			–	0.41	0.46	0.13	0.12	0.48
BAI				–	0.84^**^	0.77^**^	0.62	0.86^**^
ASI					–	0.70	0.62	0.82^**^
STAI Trait						–	0.82^**^	0.75^**^
STAI State							–	0.64
PACQ								–

*N = 30.*

** *p < 0.001*.

### Data Analyses

#### Differences in Breath-Hold Duration Between Groups (Condition A)

Two independent samples *t*-tests were conducted to compare the differences in breath-hold durations between the clinical and control groups (Table 4).

**Table 4:** *T*-test results and descriptive statistics for breath-hold (BH) tasks for clinical and control groups.

	**Group**				**95% CI for mean difference**			
	**Clinical (*n* = 16)**		**Control (*n* = 16)**					
	***M***	***SD***	***M***	***SD***		***t***	**Df**	***P***
BH p. max inhalation	44.05	18.17	53.30	18.03	−22.31, 3.82	−1.45	30	0.912
BH p. max exhalation	24.18	9.84	26.32	15.53	−11.53, 7.25	−0.465	30	0.725

The results revealed that there were no significant mean differences between the clinical participants and control participants in their breath-hold times following a maximum inhalation (*p* > 0.05). Furthermore, when comparing mean differences in breath-hold times following a passive exhalation, no significant mean differences were found between clinical participants and control participants (*p* > 0.05).

#### Physiological Differences in Response to CFI Task (Condition B)

**Figure 2:**
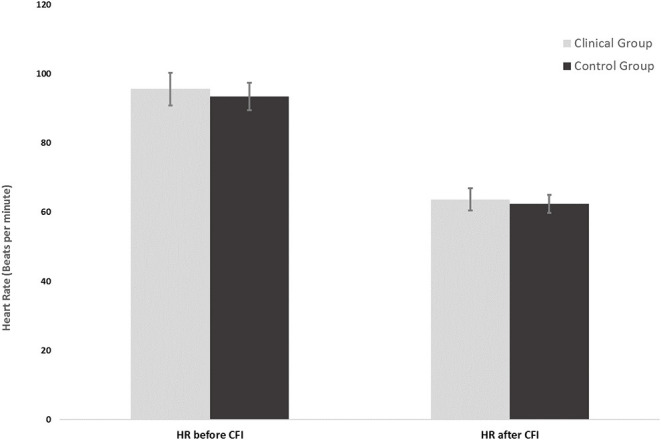
Heart rates of the clinical group (*n* = 16) and control group (*n* = 16) at the start and end of the CFI task following CO_2_ challenge.

Figure 2 shows the mean average HR measured just prior to the CFI task and the mean average HR measured upon completion of the CFI task. Simple main effects analysis showed that prior to the CFI task and upon completion of the CFI task, participants experienced a significant decrease in HR [*F*_(1, 30)_ = 58.87, *p* = 0.00, η^2^ = 0.662). However, there was no significant main effect of group, with clinical and control participants experiencing similar reductions in HR as a result of the CFI task [*F*_(1, 30)_ = 0.127, *p* = 0.724, η2 = 0.007].

#### Physiological Differences in Response to the CO_2_ Challenge (Condition C)

Table 5 shows the mean RR measured 30 s before the CO_2_ challenge (Time 1) and the mean RR measured 30 s after the CO_2_ challenge (Time 2). There was no significant interaction between the effects of group and CO_2_ challenge task on participants' RR [*F*_(1, 30)_ = 0.057, *p* = 0.814, η2 = 0.002]. Simple main effects analysis showed that between 30 s prior to the CO_2_ challenge (Time 1) and 30 s following the CO_2_ challenge (Time 2), participants experienced no significant increase in respiration rate [*F*_(1, 30)_ = 0.079 *p* = 0.780, η2 = 0.003]. No significant differences between respiration rates were observed between the clinical and control groups [*F*_(1, 30)_ = 1.062, *p* = 0.311, η2 = 0.034].

**Table 5:** Descriptive statistics for time 1 and time 2 of the CO_2_ challenge.

	**Group**			
	**Clinical (*n* = 16)**		**Control (*n* = 16)**	
	***M***	***SD***	***M***	***SD***
RR Time 1	14.09	3.52	13.29	4.18
RR Time 2	14.48	4.16	13.89	4.53
HR Time 1	92.33	15.13	85.83	12.72
HR Time 2	90.91	14.76	89.11	13.73

Furthermore, there was no significant interaction found between the effects of group and CO_2_ on participants' HR [*F*_(1, 30)_ = 0.805, *p* = 0.377, η2 = 0.026]. Simple main effects analysis showed that between 30 s prior to the CO_2_ challenge (Time 1) and 30 s following the CO_2_ challenge (Time 2), participants experienced no significant change in HR [*F*_(1, 30)_ = 0.497, *p* = 0.486, η2 = 0.016]. In addition, no significant differences between HRs were observed between the clinical and control groups [*F*_(1, 30)_ = 0.130, *p* = 0.721, η2 = 0.004].

#### Physiological Response Pre- and Post-CO_2_ Administered Prior to CFI (Condition D)

Table 6 shows the mean HRs and respiration rates for participants before and after the CO_2_ challenge. A mixed ANOVA was conducted to compare the clinical and control groups in terms of the effect of the CO_2_ challenge task and group on participants' RR and HR.

**Table 6:** Descriptive statistics for heart rate and respiration rate before and after CO_2_ challenge.

	**Group**			
	**Clinical (*n* = 16)**		**Control (*n* = 16)**	
	***M***	***SD***	***M***	***SD***
RR before CO_2_	15.80	3.60	15.93	3.03
RR after CO_2_	14.74	3.61	14.83	3.36
HR before CO_2_	88.95	15.22	89.48	8.93
HR after CO_2_	91.16	15.11	90.96	12.31

There was no significant interaction between the effects of group and CO_2_ challenge task on participants' RR [*F*_(1, 30)_ = 0.002, *p* = 0.966, η2 = 0.000]. Simple main effects analysis showed that between 30 s prior to the CO_2_ challenge (Time 1) and 30 s following the CO_2_ challenge (Time 2), participants experienced no significant increase in respiration rate [*F*_(1, 30)_ = 4.381, *p* = 0.045, η^2^ = 0.127]. No significant differences between respiration rates were observed between the panic and normal groups [*F*_(1, 30)_ = 0.011, *p* = 9.19, η^2^ = 0.000].

No significant interaction was found between the effects of group and CO_2_ on participants' HR [*F*_(1, 30)_ = 0.074, *p* = 0.788, η2 = 0.002]. Simple main effects analysis showed that between 30 s prior to the CO_2_ challenge (Time 1) and 30 s following the CO_2_ challenge (Time 2), participants experienced no significant change in HR [*F*_(1, 30)_ = 1.861, *p* = 0.183, η^2^ = 0.058]. In addition, no significant differences between HRs were observed between the clinical and control groups [*F*_(1, 30)_ = 0.001, *p* = 0.970, η^2^ = 0.000].

#### Physiological Response Before and After the CO_2_ + CFI Task (Condition D)

Figure 2 shows the mean HR at the start of the CFI following the CO_2_ challenge and the mean HR at the completion of the CFI task. A mixed ANOVA examined the effect of CFI following the CO_2_ challenge on participants' HR and investigated whether differences between groups were observed. There was no significant interaction between the effects of group and CFI on participants' HR [*F*_(1, 30)_ = 0.222, *p* = 0.641, η^2^ = 0.007). Simple main effects analysis demonstrated that between Time 1 (start of CFI task following CO_2_ challenge) and Time 2 (end of CFI task), participants experienced a significant HR reduction [*F*_(1, 30)_ = 58.878, *p* < 0.01, η^2^ = 0.662]. This was a very large effect size. However, no significant differences between the clinical and control groups were observed [*F*_(1, 30)_ = 0.127, *p* = 0.724, η^2^ = 0.004].

#### Psychological Measures and the Effects of CO_2_ and CFI

The Mann-Whitney U test was used to examine differences in the psychological measures collected across the three time periods. The PD group had significantly higher scores than the control group on all psychological measures at Time 1 (pretest), Time 2 (CO_2_), and Time 3 (CFI after CO_2_) (*p* < 0.05), with the exception of the Discomfort Intolerance Scale (DIS) (*p* = 0.09). Table 7 provides the means for all self-reported psychological measures taken at Time 1 (pretest), Time 2 (CO_2_), and Time 3 (CFI after CO_2_).

**Table 7:** Medians, minimum, maximum and interquartile ranges for psychological measures at time 1, time 2, and time 3.

	**Mdn**	**Min**	**Max**	**Interquartile range**
**Acute Panic Inventory**				
Time 1 (Pre-test) Time 2 (CO_2_ challenge) Time 3 (CO_2_ with CFI)	2.5 11.5 1	0 0 0	28 45 39	6 17.25 6.5
**Anxiety Sensitivity Index**				
Time 1 (Pre-test) Time 2 (CO_2_ challenge) Time 3 (CO_2_ with CFI)	21.5 21.5 10	0 3 0	59 63 63	19 23 22.5
**Beck Anxiety Inventory**				
Time 1 (Pre-test) Time 2 (CO_2_ challenge) Time 3 (CO_2_ with CFI)	11 17 3	0 2 0	47 60 62	28.1 27.25 9
**Panic Cognitions Questionnaire**				
Time 1 (Pre-test) Time 2 (CO_2_ challenge) Time 3 (CO_2_ with CFI)	17 7 0	0 0 0	45 68 69	27.75 18.25 5.5
**State Anxiety Inventory**				
Time 1 (Pre-test) Time 2 (CO_2_ challenge) Time 3 (CO_2_ with CFI)	38 44.5 32	21 24 9	71 79 66	17.5 22 14.5
**VAS—Participant**				
Time 1 (Pre-test) Time 2 (CO_2_ challenge) Time 3 (CO_2_ with CFI)	7 6 2	0 0 0	9 10 8	5.38 6.13 3.13
**VAS—Researcher**				
Time 1 (Pre-test) Time 2 (CO_2_ challenge) Time 3 (CO_2_ with CFI)	7.25 7 2	0 1 0	10 10 7.5	4.75 6.25 2.75

*N = 32 (group 1: n = 16; group 2: n = 16)*.

All anxiety measures including the ASI, API, STAI, PACQ, BAI, VAS-P, and VAS-R were lower at Time 3 (CO_2_ with CFI) compared to Time 1 (baseline) with the exception of API. This makes sense as control participants scored lower on the API, as they did not experience a PA in response to the CO_2_ challenge. Furthermore, all participants reported a significant decrease in anxiety symptoms across all of the measures between Time 2 (CO_2_ Challenge) and Time 3 (CO_2_ with CFI). The findings of this study lend support to the application of the diving response and CFI in reducing panic cognitions and symptoms of anxiety and panic. Furthermore, these results indicate some promise in terms of the utility of CFI in assisting with the management and reduction of anxiety and panic symptoms.

## Discussion

The current study aimed to investigate the immediate effects of breath holding and CO_2_ challenge on panic symptoms based on Klein's theory of false suffocation alarm, the principal findings of the current investigation do not support Klein's false suffocation alarm theory, which suggests that the brain's suffocation detector incorrectly signals a lack of useful air and increases vulnerability to false suffocation alarms and PAs. The study findings indicate that there were no significant differences in breath-hold durations among the clinical and control participants. Overall, the CO_2_ challenge evoked anxiety and panic symptoms as self-reported by clinical participants, and the CFI task demonstrated anxiolytic effects by reducing heart rate (HR), as well as self-reported symptoms of anxiety and panic in both the clinical and control groups. The findings of this preliminary study revealed that there was no significant difference in breath-hold (BH) ability between the clinical and control groups. While the means of the BH durations in both the passive exhalation and the maximum deep inhalation BH tasks were slightly lower for the clinical group than for the control group, these differences were not significant. One possible explanation for this finding may have been the relatively small sample size that comprised this study. Previous research that has tested this hypothesis has used different methodologies and yielded varied findings (13, 43–46). The varied results found in previous studies may be explained by many of the studies comprising small, heterogeneous samples, diverse inclusion and exclusion criteria, and different criteria for assessing panic attacks (PAs). Lung capacity decreasing with age is also another known factor that may reduce breath-hold (BH) ability. Despite lung volumes being different, there are no gender differences in BH ability (47).

Furthermore, these findings do not support the BH challenge as a potential marker for hypersensitivity to CO_2_ and susceptibility to CO_2_-induced panic. Hypersensitivity to CO_2_ may be more notable in the PD respiratory subtype (4, 48–53). The small sample in this study and our recruitment method (general rather than selecting subgroups) precluded subgroup analysis. Respiratory symptoms may play a role in both PAs and CO_2_-induced panic (44). Their study reported that with a single breath of 35% CO_2_/65% O_2_ inhalation, participants with PD reported significantly stronger symptoms of panic and anxiety than the control group. These findings were in line with those of Griez et al. (2) and Perna et al. (11). The results of the current study support this hypothesis and found that both clinical and control participants demonstrated a significant bradycardic effect (a drop of ~30–35 beats per minute) following the CFI task. This is in line with previous research that has demonstrated that the diving response (DR) elicits a strong autonomic response characterized by a pronounced HR reduction and blood centralization to the organs that are most in need of oxygen (i.e., heart, lungs and brain) (25, 34, 54–57). The findings of the current study suggest that the DR is a powerful physiological adaptation that is innate to all humans. This is consistent with previous research that has found that the DR is augmented by the CFI task or by facial cooling (30, 36, 58).

Furthermore, our study found no significant differences between the clinical and control groups in HR in response to CO_2_. Previous research has yielded mixed results, with some CO_2_ studies reporting an increase in HR (59–63) and some reporting a decrease or no change in HR (64).

In addition, this study reported no significant difference in respiration rates between the clinical and control groups. Although previous research examining the respiration rate (RR) after CO_2_ challenge has yielded mixed results, our findings lend support to those of existing research (6, 65–67), which revealed no significant differences in respiration rates following CO_2_ inhalation. Contrary to the findings of van den Hout and Griez's study, the CO_2_ challenge did not induce a significant increase in RR in clinical and control participants (12). Respiratory rate may not be the best measure of respiratory response to CO_2_ but rather the increase in respiratory tidal volume. Previous studies have reported that 50% of clinical panic participants describe difficulties with taking a deep inhalation of CO_2_ and feeling breathlessness (68). This difficulty was also observed with the clinical group in this study, with some participants reporting discomfort in breathing and with the bad odor. Hence, it is plausible that the full effects of CO_2_ were not observed, as some participants may not have taken full inhalation of CO_2._ For the 35% CO_2_ test to be valid, a participant needs to inhale at least 80% of their vital capacity (66).

With regard to BH durations, our findings were not in line with the findings of Asmundson and Stein's, who found that patients diagnosed with PD had significantly shorter BH durations than healthy participants (43). Our findings were not in line with Klein's theory of the suffocation false alarm theory (6) and the findings of Asmundson and Stein, who suggested that participants with PD terminate their BH earlier to avoid activation of the suffocation alarm (43). A relatively small sample size of the clinical group may have been a plausible explanation for this finding. Future research on BH durations between clinical and control groups should look at investigations with a larger sample size that is adequately powered.

The current findings are consistent with the finding from a study that showed no significant differences in HR changes between panic patients and controls following the CO_2_ challenge, even though there was a trend for the heart rate increasing (69). It is well-established in the literature that CO_2_-induced inhalation elicits a sudden increase in ventilation accompanied by a surge of anxiety that mimics a PA (6, 66) and triggers arousal of the conditioned fear response in panic patients (70, 71). It was demonstrated that panic patients who experienced CO_2_-induced PAs showed HR responses to CO_2_ that were significantly greater than those of non-panic patients, perhaps reflecting greater cardiac sympathetic stimulation by CO_2_ (63).

Limitations of this study included recruitment challenges, and the extensive list of exclusion criteria for individuals to be eligible to participate in the study which limited the sample size and matching of participants. A pragmatic approach was pursued in an attempt to match participants for age and gender however this was not possible due to recruitment challenges. There were also some difficulties noted with the breathing apparatus for the CO_2_ challenge and with the provocation method used. Amongst some of the challenges some participants reported included: disliking the taste of the CO_2_ gas, feeling anxious or panicky when doing the task, difficulty with inhaling as deeply as instructed, whilst others were not able to hold their breath with the inhaled gas mixture for a period of 4 s before exhaling it, as instructed. Given that to be considered a valid test, participants need to inhale at least 80% of their vital capacity of CO_2_ (66). Our results may have been impacted by the inability of some participants to achieve this. Given the brevity of the task, it was not anticipated that participants would experience difficulty in carrying out the CO_2_ challenge as per instructions. Future studies should emphasize to participants the importance of maximum inhalation and holding their breath for 4 s. Another important limitation was the variability in participants' heart rate and respiration rate changes in response to the CO_2_ challenge, which made data interpretation difficult when comparing 30 s of physiological data 30 s prior to the CO_2_ challenge to 30 s following the CO_2_ challenge. Nonetheless, when data were examined on a case-by-case basis, a trend was depicted, characterized by a more elevated RR and HR in the clinical participants in response to the CO_2_ challenge compared to the control group.

It is noteworthy that by activating the diving response and subsequently reducing one's heart rate, one may achieve reductions in physiological and cognitive symptoms of panic and potentially in CO_2_ sensitivity. This study demonstrated that the CFI task was able to reduce anxiety and panic symptoms induced by the CO_2_ challenge. One of the most frightening symptoms reported by sufferers of panic disorder is heart racing or pounding. Hence, reducing the heart rate and autonomic sympathetic nervous system arousal may have a positive impact on self-reported anxiety. Another common fear-associated symptom reported by panic sufferers is the feeling of suffocation and dyspnea. In contrast, when the diving response is activated, it exerts an oxygen-conserving effect that extends breath-holding time with the aim of assisting the survival of the organism. Hence, CFI may prove to be an effective treatment for panic disorder and other anxiety disorders. Furthermore, the diving response can be easily activated with cold moisture (i.e., ice packs), making it an easily administered treatment. Further investigations are warranted to explore the anxiolytic effects induced by the activation of the diving response.

## Data Availability Statement

The raw data supporting the conclusions of this article will be made available by the authors, without undue reservation.

## Ethics Statement

The studies involving human participants were reviewed and approved by Swinburne University Human Research Ethics Committee. The participants provided their written informed consent to participate in this study.

## Author Contributions

PK provided the conceptualization, methodology, analysis, investigation, writing, reviewing and editing, and funding of the research. MK and MS provided supervision and review. AN and RF provided the review and editing. All authors contributed to the article and approved the submitted version.

## Conflict of Interest

The authors declare that the research was conducted in the absence of any commercial or financial relationships that could be construed as a potential conflict of interest.

## Publisher's Note

All claims expressed in this article are solely those of the authors and do not necessarily represent those of their affiliated organizations, or those of the publisher, the editors and the reviewers. Any product that may be evaluated in this article, or claim that may be made by its manufacturer, is not guaranteed or endorsed by the publisher.
